# Comparison of *dkgB*-linked intergenic sequence ribotyping to DNA microarray hybridization for assigning serotype to *Salmonella enterica*

**DOI:** 10.1111/1574-6968.12010

**Published:** 2012-10-19

**Authors:** Jean Guard, Roxana Sanchez-Ingunza, Cesar Morales, Tod Stewart, Karen Liljebjelke, JoAnn Kessel, Kim Ingram, Deana Jones, Charlene Jackson, Paula Fedorka-Cray, Jonathan Frye, Richard Gast, Arthur Hinton

**Affiliations:** 1 Agricultural Research Service, U.S. Department of AgricultureAthens, GA, USA; 2 Agricultural Research Service, U.S. Department of AgricultureBeltsville, MD, USA

**Keywords:** epidemiology, food safety, single nucleotide polymorphism

## Abstract

Two DNA-based methods were compared for the ability to assign serotype to 139 isolates of *Salmonella enterica* ssp. I. Intergenic sequence ribotyping (ISR) evaluated single nucleotide polymorphisms occurring in a 5S ribosomal gene region and flanking sequences bordering the gene *dkgB*. A DNA microarray hybridization method that assessed the presence and the absence of sets of genes was the second method. Serotype was assigned for 128 (92.1%) of submissions by the two DNA methods. ISR detected mixtures of serotypes within single colonies and it cost substantially less than Kauffmann–White serotyping and DNA microarray hybridization. Decreasing the cost of serotyping *S. enterica* while maintaining reliability may encourage routine testing and research.

## Introduction

Serotyping of *Salmonella enterica* ssp. I is the basis of national and international surveillance and communications, it facilitates determining associations between the pathogen and sources, and it gives some guidance in regards to preventing transmission (P. Fields, pers. commun.) (Foodnet, 2011). The historical method used to serotype *S. enterica* is the antibody-based Kauffman–White (KW) scheme (Kauffman & Edwards, 1952; Brenner *et al*., 2000). Positive results generate an antigenic formula based on structural details of the H-antigen of flagella and the O-antigen of lipopolysaccharide (H- and O-antigens, respectively) (Bopp *et al*., 1999; Popoff & Le Minor, 2001).

**Fig. 1:**
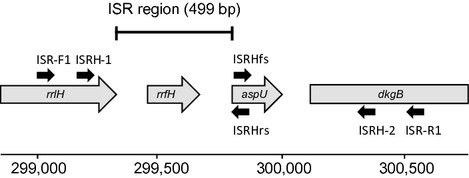
Description of the ISR region within the genome of *Salmonella enterica* serovar Enteritidis strain P125109 (GenBank AM933172). The nucleotide sequence of each ISR is serotype specific and size ranges from 257 to 530 bp. Sequence is required to assign serotype to a submission. An ISR region includes sequence from the end of the *rrlH* gene (rRNA-23S ribosomal gene) and the start of the *aspU* (tRNA-Asp) gene that is adjacent to the *dkgB* gene.

A major advantage of DNA analysis is that it is not impacted by variable expression of cell-surface antigens as are antibody-based agglutination assays like the KW scheme. Major obstacles to genome typing of *S. enterica* becoming broadly available include expense, the need for highly specialized equipment, and in some cases, sophisticated bioinformatics (Wattiau *et al*., 2011). A DNA-based method for assigning serotype to *S. enterica* at comparatively low cost and with readily accessible laboratory equipment commonly used for culturing and conducting the polymerase chain reaction (PCR) would be beneficial. A discrete region within *S. enterica* ssp. I was previously shown to differentiate closely related serotypes (Morales *et al*., 2006). The region of interest spans from the end of a 23S ribosomal gene, across a 5S gene and includes the last base pair preceding a tRNA *aspU* ribosomal gene neighboring *dkgB* (previously *yafB*) (Fig. 1). We wanted to know if *dkgB*-linked intergenic sequence ribotyping (ISR) would assign serotype similarly to an AOAC International certified DNA microarray hybridization method (DNAhyb) (Check & Trace by Checkpoints, Certificate 121001) (Malorny *et al*., 2003; Wattiau *et al*., 2008; Madajczak & Szych, 2010). The set of isolates examined were previously assigned a KW serotype and this historical information is included.

## Materials and methods

### *Salmonella enterica* submissions included for analysis

The investigators providing the submissions listed in Table 1 reported serotype. In this laboratory, cultures were streaked on brilliant green (BG) agar (Acumedia; Neogen Corporation, Lansing, MI) and incubated for 24–48 h at 37 °C to obtain well-separated large colonies. One colony was then transferred to brain heart infusion (BHI) broth (Acumedia) and incubated for 16 h at 37 °C with shaking. For submissions that later appeared to have mixed cultures and for those with disagreement between methods, agglutination reactions for single colonies using commercially available absorbed antisera (Difco, BD, Franklin Lakes, NJ) were carried out, and in some cases, isolates were submitted for serotyping (Silliker, South Holland, IL). Thus, single colonies were processed by both ISR and DNAhyb (Check & Trace, Check Points, Wageningen, the Netherlands). In cases of disagreement between methods, a maximum of 10 well-isolated colonies were selected from agar plates and then transferred to BHI broth for individual analysis.

**Table 1:** Serotype of *Salmonella enterica* ssp. I as determined by the KW scheme, DNAhyb, and *dkgB-*linked ISR

Accession Number	KW scheme	DNAhyb	ISR	ISR size	Supplier	Source	Locality	Category
*(a) Submissions with agreement between DNA-based genomic methods DNAhyb and ISR, and with no conflict to serotype as reported using the KW scheme [115]*								
21027	Enteritidis	Enteritidis 2994.G	Enteritidis	499	ESQRU, ARS, USDA	Mouse spleen	Northeast US	TP
21046	Enteritidis	Enteritidis 2994.G	Enteritidis	499	ESQRU, ARS, USDA	Mouse spleen	Northeast US	TP
22079	Enteritidis	Enteritidis 2994.G	Enteritidis	499	UC	Creek	California	TP
23023	Pullorum	Gallinarum Pullorum 2978.H	Pullorum	361	ESQRU, ARS, USDA	Unknown	Unknown	TP
24018	Newport	Newport 12427	Newport	498	NVSL	Unknown	Unknown	TP
25001	Agona	Agona 7205	Agona	498	SGSC	Unknown	Peru	TP
25006	Choleraesuis	Choleraesuis or Paratyphi C 13545	Cholerasuis	499	SGSC	Unknown	Thailand	TP
25012	Dublin	Dublin (probability 99.92%) 2488	Dublin	499	SGSC	Cattle	Idaho	TP
25013	Dublin	Dublin (probability 99.92%) 2488	Dublin	499	SGSC	Bovine	France	TP
25021	Gallinarum	Gallinarum Gallinarum 2978	Gallinarum	498	SGSC	Human	Connecticut	TP
25026	Infantis	Infantis 9381	Infantis_2	500	SGSC	Human	North Carolina	TN
25030	Montevideo	Montevideo 6690	Montevideo_1	362	SGSC	Human	Georgia	TP
25031	Montevideo	Montevideo 6702	Montevideo_2	361	SGSC	Human	Florida	TP
25042	Paratyphi A	Paratyphi A 14413	Paratyphi A	498	SGSC	Unknown	Unknown	TP
25049	Paratyphi C	Choleraesuis or Paratyphi C 13545	Paratyphi C	395	SGSC	Human	France	TP
25052	Pullorum	Gallinarum Pullorum 2978.H	Pullorum	361	SGSC	Unknown	Germany	TP
25063	Typhi	Typhi 7241	Typhi	267	SGSC	Unknown	Dakar	TP
25064	Typhi	Typhi 7241	Typhi	267	SGSC	Unknown	Dakar	TP
26022	Cerro	Cerro 4237	Cerro	361	EMSFL, ARS, USDA	Fecal (dairy cow)	Unknown	TP
26023	Cerro	Cerro 4237	Cerro	361	EMSFL, ARS, USDA	Lung (dairy cow)	Unknown	TP
26024	Cerro	Cerro 4237	Cerro	361	EMSFL, ARS, USDA	Fecal (dairy cow)	Unknown	TP
26028	Oranienburg	Oranienburg 6717 r	Oranienburg	365	EMSFL, ARS, USDA	Fecal (dairy cow)	Unknown	TP
26029	Oranienburg	Oranienburg 6717	Oranienburg	365	EMSFL, ARS, USDA	Fecal (dairy cow)	Unknown	TP
26030	Typhimurium 5-	Typhimurium 10909	Typhimurium	498	EMSFL, ARS, USDA	Fecal (dairy cow)	Unknown	TP
26039	Montevideo	Montevideo 6702	Montevideo_3	362	EMSFL, ARS, USDA	Milk	Unknown	TN
26050	Agona	Agona 7205	Agona	498	EMSFL, ARS, USDA	Fecal (dairy cow)	Unknown	TP
26080	Agona	Agona 7205	Agona	498	EMSFL, ARS, USDA	Fecal (dairy cow)	Unknown	TP
29047	Typhimurium	Typhimurium 10909	Typhimurium	498	ESQRU, ARS, USDA	Unknown	Unknown	TP
29054	Typhimurium	Typhimurium 10909	Typhimurium	498	ESQRU, ARS, USDA	Unknown	Unknown	TP
29056	Typhimurium	Typhimurium 10909	Typhimurium	498	ESQRU, ARS, USDA	Unknown	Unknown	TP
29096	Schwarzengrund	Schwarzen. or Grumpensis 14909.B	Schwarzengrund	257	PPSPR, ARS, USDA	Scalder tank water	Georgia	TP
99113	Pullorum	Gallinarum Pullorum 2978.H	Pullorum	361	CFIA	Chicken House	Unknown	TP
99117	Gallinarum	Gallinarum Gallinarum 2978	Gallinarum	498	CFIA	Chicken House	Unknown	TP
99163	Typhimurium 5-	Typhimurium 10909	Typhimurium	498	USDA, ARS, TX	Pigeon	Unknown	TP
99164	Typhimurium 5-	Typhimurium 10909	Typhimurium	498	ESQRU, ARS, USDA	Unknown	Unknown	TP
99172	Typhimurium 5-	Typhimurium 10909	Typhimurium	498	USDA, ARS	Pigeon	Unknown	TP
100304.05	Kentucky	Kentucky 10299	Kentucky	492	PPSPR, ARS, USDA	Carcass rinse	Georgia	TP
100304.07	Kentucky	Kentucky 10299	Kentucky	492	PPSPR, ARS, USDA	Carcass rinse	Georgia	TP
100304.08	Kentucky	Kentucky 10299	Kentucky	492	PPSPR, ARS, USDA	Scalder tank foam	Georgia	TP
100304.19	1,4,[5],12:i:-	1,4,[5],12:i:- 2717	1,4,[5],12:i:-	498	PPSPR, ARS, USDA	Carcass rinse	Georgia	TP
100304.32	Typhimurium 5-	Typhimurium 10909	Typhimurium	498	PPSPR, ARS, USDA	Carcass rinse	Georgia	TP
100304.43	Heidelberg	Heidelberg 15835	Heidelberg	498	PPSPR, ARS, USDA	Carcass rinse	Georgia	TP
100304.48	Heidelberg	Heidelberg 15835	Heidelberg	498	PPSPR, ARS, USDA	Carcass rinse	Georgia	TP
100304.52	Heidelberg	Heidelberg 15835	Heidelberg	498	PPSPR, ARS, USDA	Carcass rinse	Georgia	TP
100304.57	Typhimurium 5-	Typhimurium 10909	Typhimurium	498	PPSPR, ARS, USDA	Carcass rinse	Georgia	TP
100304.63	Typhimurium 5-	Typhimurium 10909	Typhimurium	498	PPSPR, ARS, USDA	Carcass rinse	Georgia	TP
100304.69	Thompson	Thompson 14415	Thompson	259	PPSPR, ARS, USDA	Carcass rinse	Georgia	TP
100304.74	Senftenberg	Senftenberg 2156	Senftenberg	362	PPSPR, ARS, USDA	Carcass rinse	Georgia	TP
100304.75	Senftenberg	Schwarzen. or Grumpensis 14909.B	Schwarzengrund	257	PPSPR, ARS, USDA	Carcass rinse	Georgia	TP
100304.76	Senftenberg	Schwarzen. or Grumpensis 14909.B	Schwarzengrund	257	PPSPR, ARS, USDA	Carcass rinse	Georgia	TP
100304.78	Thompson	Thompson 14415	Thompson	259	PPSPR, ARS, USDA	Carcass rinse	Georgia	TP
100304.79	Thompson	Thompson 14415	Thompson	259	PPSPR, ARS, USDA	Carcass rinse	Georgia	TP
100616.101	Kentucky	Kentucky 10299	Kentucky	492	PPSPR, ARS, USDA	Carcass rinse	Georgia	TP
100616.87	1,4,[5],12:i:-	1,4,[5],12:i:- 2717	1,4,[5],12:i:-	498	PPSPR, ARS, USDA	Scalder tank foam	Georgia	TP
100616.89	Typhimurium	Typhimurium 10909	Typhimurium	498	PPSPR, ARS, USDA	Carcass rinse	Georgia	TP
100616.9	Typhimurium 5-	Typhimurium 10909	Typhimurium	498	PPSPR, ARS, USDA	Carcass rinse	Georgia	TP
100616.91	Typhimurium	Typhimurium 10909	Typhimurium	498	PPSPR, ARS, USDA	Carcass rinse	Georgia	TP
100709.01	Kentucky	Kentucky 10299	Kentucky	492	PPSPR, ARS, USDA	Carcass rinse	Georgia	TP
100709.02	Kentucky	Kentucky 10299	Kentucky	492	PPSPR, ARS, USDA	Carcass rinse	Georgia	TP
100709.03	Kentucky	Kentucky 10299	Kentucky	492	PPSPR, ARS, USDA	Carcass rinse	Georgia	TP
100709.04	Kentucky	Kentucky 10299	Kentucky	492	PPSPR, ARS, USDA	Carcass rinse	Georgia	TP
100709.05	Senftenberg	Senftenberg 2156	Senftenberg	362	PPSPR, ARS, USDA	Carcass rinse	Georgia	TP
100709.09	Heidelberg	Heidelberg 15835	Heidelberg	498	PPSPR, ARS, USDA	Scalder tank foam	Georgia	TP
100721.01-2	1,4,[5],12:i:-	1,4,[5],12:i:- 2717	1,4,[5],12:i:-	498	PPSPR, ARS, USDA	Scalder tank water	Georgia	TP
100721.02	Infantis	Infantis 9381	Infantis_1	500	PPSPR, ARS, USDA	Carcass rinse	Georgia	TP
100721.05	Typhimurium	Typhimurium 10909	Typhimurium	498	PPSPR, ARS, USDA	Carcass rinse	Georgia	TP
100723.09	Enteritidis	Enteritidis 2994.G	Enteritidis	499	PPSPR, ARS, USDA	Carcass rinse	Georgia	TP
100304.10	1,4,[5],12:i:-	1,4,[5],12:i:- 2717	1,4,[5],12:i:-	498	PPSPR, ARS, USDA	Carcass rinse	Georgia	TP
100304.50	Heidelberg	Heidelberg 15835	Heidelberg	498	PPSPR, ARS, USDA	Carcass rinse	Georgia	TP
100709.10	Thompson	Thompson 14415	Thompson	259	PPSPR, ARS, USDA	Scalder dip tank foam	Georgia	TP
100723.10	Enteritidis	Enteritidis 2994.G	Enteritidis	499	PPSPR, ARS, USDA	Scalder dip tank foam	Georgia	TP
100304.58-2	Kentucky	Kentucky 10299	Kentucky	492	PPSPR, ARS, USDA	Scalder tank foam	Georgia	TP
100304.62-2	Typhimurium 5-	Typhimurium 10909	Typhimurium	498	PPSPR, ARS, USDA	Scalder dip tank foam	Georgia	TP
100616.86-2	Typhimurium	Typhimurium 10909	Typhimurium	498	PPSPR, ARS, USDA	Carcass rinse	Georgia	TP
100723.01-2	Kentucky	Kentucky 10299	Kentucky	492	PPSPR, ARS, USDA	Carcass rinse	Georgia	TP
100723.14-2	Schwarzengrund	Schwarzen. or Grumpensis 14909.B	Schwarzengrund	257	PPSPR, ARS, USDA	Carcass rinse	Georgia	TP
100723.02-2	Heidelberg	Heidelberg 15835	Heidelberg	498	PPSPR, ARS, USDA	Scalder tank foam	Georgia	TP
25010	Derby	Derby 50	Derby	498	SGSC	Swine	Minnesota	TP
25032	Muenchen	Muenchen 11942	Muenchen	404	SGSC	Unknown	Unknown	TP
25039	Panama	Panama 14909	Panama	362	SGSC	Unknown	Italy	TP
26063	Mbandaka	Mbandaka 11813.C	Mbandaka	499	EMSFL, ARS, USDA	Fecal (dairy cow)	Unknown	TP
26066	Mbandaka	Mbandaka 11813.C	Mbandaka	499	EMSFL, ARS, USDA	Fecal (dairy cow)	Unknown	TP
100616.100	Rough	Infantis 9381	Infantis_1	500	PPSPR, ARS, USDA	Carcass rinse	Georgia	TP
100616.102	Rough	Enteritidis 2866.G	Enteritidis	499	PPSPR, ARS, USDA	Scalder tank water	Georgia	TP
100616.84	Rough	Typhimurium 10909	Typhimurium	498	PPSPR, ARS, USDA	Carcass rinse	Georgia	TP
100616.97	Rough	Typhimurium 10909	Typhimurium	498	PPSPR, ARS, USDA	Carcass rinse	Georgia	TP
100616.99	Rough	Typhimurium 10909	Typhimurium	498	PPSPR, ARS, USDA	Carcass rinse	Georgia	TP
100709.06	Rough	Senftenberg 2156	Senftenberg	362	PPSPR, ARS, USDA	Carcass rinse	Georgia	TP
100709.07	Rough	1,4,[5],12:i:- 2717	1,4,[5],12:i:-	498	PPSPR, ARS, USDA	Carcass rinse	Georgia	TP
100709.12	Rough	Infantis 9381	Infantis_1	500	PPSPR, ARS, USDA	Carcass rinse	Georgia	TP
100723.04	Rough	Kentucky 10299	Kentucky	492	PPSPR, ARS, USDA	Carcass rinse	Georgia	TP
100723.08	Auto agglutinator	Heidelberg 15835	Heidelberg	498	PPSPR, ARS, USDA	Scalder tank foam	Georgia	TP
100723.12	Rough	Senftenberg 2156 r	Senftenberg	362	PPSPR, ARS, USDA	Carcass rinse	Georgia	TP
100723.15	Rough	Schwarzen. or Grumpensis 14909.B	Schwarzengrund	257	PPSPR, ARS, USDA	Scalder tank water	Georgia	TP
25046	Salmonella (B)	Abony 5325	Abony	498	SGSC	Water	United Kingdom	TP
25050	Salmonella (C1)	Oranienburg 6717 r	Oranienburg	361	SGSC	Human	France	TP
29063	Salmonella (C1)	Newport 13444	Newport_1	499	ESQRU, ARS, USDA	Unknown	Unknown	TP
29065	Salmonella (C1)	Oranienburg 6717 r	Oranienburg	365	ESQRU, ARS, USDA	Unknown	Unknown	TP
29066	Salmonella (B)	Typhimurium 10909	Typhimurium	498	ESQRU, ARS, USDA	Unknown	Unknown	TP
29067	Salmonella (D)	Enteritidis 2994.G	Enteritidis	499	ESQRU, ARS, USDA	Unknown	Unknown	TP
25004	No O or H antigen	Anatum 15087	Anatum	499	SGSC	Swine	Minnesota	TP
25037	Newport	Newport 13519	Newport_2	395	SGSC	Human	Mexico	TP
25038	Newport	Newport 14539	Newport_3	498	SGSC	Snake	Massachusetts	TP
101116-10*	Fresno (D2)	Genovar 5216	UN0019 (D1)	258	Breeder farm	Chickens	Alabama or Tennessee	TP
101116-12*	Fresno (D2)	Genovar 5216	UN0019 (D1)	258	Breeder farm	Chickens	Alabama or Tennessee	TP
25005	Choleraesuis	Genovar 14861	UN0009 (C1)	361	SGSC	Unknown	Switzerland	TP
25007	Choleraesuis	Genovar 11439 N	UN0010 (C1)	498	SGSC	Unknown	Australia	TP
25017	Enteritidis	Genovar 6660	UN0002 (D1)	360	SGSC	Unknown	Brazil	TP
25019*	Enteritidis	Genovar 2370	UN0003 (-)	498	SGSC	Unknown	Switzerland	TP
25009	Derby	Genovar 6176	UN0022 (B)	498	SGSC	Avian	Oklahoma	TP
25014	Dublin	Genovar 5324	UN0012 (D1)	498	SGSC	Unknown	Thailand	TP
25027	Infantis	Genovar 14892	UN0023 (C1)	499	SGSC	Unknown	Senegal	TP
25034	Muenchen	Genovar 13646	UN0036 (C2C3)	258	SGSC	Human	France	TP
99167	Typhimurium 5-	Typhimurium 10909	Typhimurium 5-	395	USDA, ARS	Pigeon	Unknown	TN
99168	Typhimurium 5-	Typhimurium 10909	Typhimurium 5-	395	USDA, ARS	Pigeon	Unknown	TN
*(b) Submissions with agreement between DNAhyb and ISR, but disagreeing with serotype as reported using the KW scheme [13]*								
25040	Panama (D1)	Javiana 12917	Javiana (D1)	367	SGSC	Human	North Carolina	TP
25041	Panama (D1)	Javiana 12917	Javiana (D1)	367	SGSC	Human	North Carolina	TP
25035	Muenchen (C2)	Manhattan 11706	Manhattan (C2)	396	SGSC	Human	North Carolina	TP
25051-1†‡	Pullorum (D1)	Oranienburg 6717 r	Oranienburg (-)	361	SGSC	Unknown	Unknown	TP
26026	Mbandaka (C1)	Tennessee 2108	Tennessee (C1)	258	EMSFL, ARS, USDA	Fecal (dairy cow)	Unknown	TP
100304.58-1†‡	Kentucky (C2C3)	Typhimurium 10909	Typhimurium (B)	498	PPSPR, ARS, USDA	Scalder tank foam	Georgia	TP
100304.62-1†‡	Typhimurium 5- (B)	Schwarzen. or Grumpensis 14909.B	Schwarzengrund (B)	257	PPSPR, ARS, USDA	Scalder dip tank foam	Georgia	TP
100616.86-1†‡	Typhimurium (B)	Infantis 9381	Infantis_1 (C1)	500	PPSPR, ARS, USDA	Carcass rinse	Georgia	TP
100616.98-1†	Typhimurium 5- (B)	Kentucky 10299	Kentucky (C2C3)	492	PPSPR, ARS, USDA	Carcass rinse	Georgia	TP
100721.01-1†‡	1,4,[5],12:i:- (B)	Kentucky 10299	Kentucky (C2C3)	492	PPSPR, ARS, USDA	Scalder tank water	Georgia	TP
100304.53	Senftenberg (B)	Typhimurium 10909	Typhimurium (B)	498	PPSPR, ARS, USDA	Scalder tank water	Georgia	TP
25011*	Derby (B)	Genovar 5160	UN0025 (C2C3)	498	SGSC	Turkey	Pennsylvania	TP
25048*	Paratyphi C (C1)	Genovar 14375	UN0024 (B)	499	SGSC	Unknown	France	TP
*(c) Submissions with disagreement between DNAhyb and ISR, but with DNAhyb in agreement with serotype as reported by the KW scheme [11]*								
25036	Newport	Newport 13444	UN0034	498	SGSC	Human	North Carolina	FP
25043-2	Paratyphi B (B)	Paratyphi B (possibly Java) 13383	UN0015	498	SGSC	Unknown	Unknown	FP
25044	Paratyphi B (B)	Paratyphi B (possibly Java) 13383	UN0011	499	SGSC	Food	Middle East	FP
25051-2	Pullorum (D1)	Gallinarum Pullorum 2978.H	UN0008	530	SGSC	Unknown	Unknown	FP
25057	Schwarzengrund	Schwarzen. or Grumpensis 14909.B	UN0006	365	SGSC	Unknown	Scotland	FP
26078	Kentucky	Kentucky (10791) 6	UN0028	499	EMSFL, ARS, USDA	Fecal (dairy cow)	Unknown	FP
101116-14	Javiana	Javiana 12917	UN0007	361	Breeder farm	Chickens	Alabama or Tennessee	FP
25043-1†‡	Paratyphi B (B)	Paratyphi B (possibly Java) 13383	Javiana (D1)	367	SGSC	Unknown	Unknown	TN
100723.01-1†‡	Heidelberg (B)	Heidelberg 15835	Kentucky (C2C3)	498	PPSPR, ARS, USDA	Carcass rinse	Georgia	TN
100723.02-1†‡	Heidelberg (B)	Heidelberg 15835	Kentucky (C2C3)	492	PPSPR, ARS, USDA	Scalder tank foam	Georgia	TN
100723.14-1†‡	Kentucky (C2C3)	Kentucky 10299	Schwarzengrund (B)	492	PPSPR, ARS, USDA	Carcass rinse	Georgia	TN

* O-antigen immunoreactivity group of KW scheme did not match ISR results, but O-antigens D1 and D2 are cross-reactive.

† Submissions with hyphenation with numerical extension (-1) were classified as potentially containing a mixture of at least two serotypes as evidenced by forward/reverse ISR sequences that did not match when first evaluated. If a second serotype was isolated, it has extension -2 and is listed elsewhere in the table.

‡ Mixtures of serotypes were confirmed by processing at least 10 CFU.

### Determination of ISR

The locations where primers hybridize the reference genome in *S. enterica* ssp. I Enteritidis strain P125109 are shown in Fig. 1. Forward (ISR-F1) and reverse (ISR-R1) primers incorporate the rRNA-23S ribosomal ribose nucleic acid (RNA) region neighboring *dkgB* (previously known as *yafB*). Reference genomes and primers used in these analyses are listed in Tables 2 and 3. Primers ISR-F1 and ISR-R1 replaced previously published primers ISRH-1 and ISRH-2 (Morales *et al*., 2006).

**Table 2:** Reference ISR sequences available in public databases for *Salmonella enterica* ssp. I

Serotype designation	ISR size (bp)	Refseq	GenBank accession
(a) Genome sequences used to obtain ISRs for *Salmonella enterica* ssp. I at The National Center for Biotechnology Information (http://www.ncbi.nlm.nih.gov/)			
4,[5],12:i:- str. CVM23701	498	NZ_ABAO00000000	ABAO00000000
Agona str. SL483	498	NZ_ABEK00000000	ABEK00000000
Choleraesuis str. SC-B67	499	NC_006905	AE017220
Dublin str. CT_02021853	499	NZ_ABAP00000000	ABAP00000000
Heidelberg str. SL476	498	NC_011083	CP001120
Heidelberg str. SL486	498	NZ_ABEL00000000	ABEL00000000
Javiana str. GA_MM04042433	367	NZ_ABEH00000000	ABEH00000000
Kentucky str. CDC 191	492	NZ_ABEI00000000	ABEI00000000
Kentucky str. CVM29188	492	NZ_ABAK00000000	ABAK00000000
Newport str. SL254	492	NC_011080	CP000604
Newport str. SL317	492	NZ_ABEW00000000	ABEW00000000
Paratyphi A str. ATCC 9150	498	NC_006511	CP000026
Paratyphi B SPB7	498	NC_010102.1	CP000886
Paratyphi C RKS4594	395	NC_012125.1	CP000857
Saintpaul str. SARA23	498	NZ_ABAM00000000	ABAM00000000
Saintpaul str. SARA29	498	NZ_ABAN00000000	ABAN00000000
Schwarzengrund str. CVM19633	267	NC_011094	CP001127
Schwarzengrund str. SL480	267	NZ_ABEJ00000000	ABEJ00000000
Typhi Ty2	267	NC_004631	AE014613
Typhi str. CT18	267	NC_003198	AL513382
Typhimurium LT2	498	NC_003197	AE006468
Virchow str. SL491	499	Not yet completed	na
(b) Genome sequences used to obtain ISRs for *Salmonella enterica* ssp. I available at The Sanger Institute (http://www.sanger.ac.uk/Projects/Salmonella/)			
Enteritidis PT4 NCTC 13349	499	NC_011294.1	AM933172.1
Gallinarum 287/91 NCTC 13346	498	NC_011274.1	AM933173.1
Hadar	498	NA*	NA
Infantis	500	NA	NA
Typhimurium DT104 NCTC 13348	498	NA	NA
Typhimurium DT2	498	NA	NA
Typhimurium SL1344 NCTC 13347	498	NA	NA
Typhimurium D23580	498	NA	NA
(c) ISR sequences for *Salmonella enterica* ssp. I submitted to the National Center for Biotechnology Information†			
Schwarzengrund_2	257	In process	In process
Cerro	361	In process	JN105120
Infantis_1	500	In process	JN105121
Oranienburg	365	In process	JN105122
Pullorum	361	In process	JN105123
Senftenberg	362	In process	JN105124
Thompson	259	In process	JN105125
Tennessee	258	BankIt1458394 SEQ_018	JN092310
Mbandaka	499	BankIt1458394 SEQ_030	JN092322
Montevideo_1	362	BankIt1458394 SEQ_031	JN092323
Montevideo_2	361	BankIt1458394 SEQ_032	JN092324
Montevideo_3	362	BankIt1458394 SEQ_033	JN092325
Manhatten	258	BankIt1458394 SEQ_036	JN092328
UN0001	404	BankIt1458394 SEQ_001	JN092293
UN0002	360	BankIt1458394 SEQ_002	JN092294
UN0003	498	BankIt1458394 SEQ_003	JN092295
UN0004	362	BankIt1458394 SEQ_004	JN092296
UN0005	361	BankIt1458394 SEQ_005	JN092297
UN0006	365	BankIt1458394 SEQ_006	JN092298
UN0007	361	BankIt1458394 SEQ_007	JN092299
UN0008	530	BankIt1458394 SEQ_008	JN092300
UN0009	361	BankIt1458394 SEQ_009	JN092301
UN0010	498	BankIt1458394 SEQ_010	JN092302
UN0011	499	BankIt1458394 SEQ_011	JN092303
UN0012	498	BankIt1458394 SEQ_012	JN092304
UN0013	498	BankIt1458394 SEQ_013	JN092305
UN0014	395	BankIt1458394 SEQ_014	JN092306
UN0015	489	BankIt1458394 SEQ_015	JN092307
UN0016	396	BankIt1458394 SEQ_016	JN092308
UN0017	395	BankIt1458394 SEQ_017	JN092309
UN0019	258	BankIt1458394 SEQ_019	JN092311
UN0021	499	BankIt1458394 SEQ_021	JN092313
UN0022	498	BankIt1458394 SEQ_022	JN092314
UN0023	499	BankIt1458394 SEQ_023	JN092315
UN0024	499	BankIt1458394 SEQ_024	JN092316
UN0025	498	BankIt1458394 SEQ_025	JN092317
UN0026	498	BankIt1458394 SEQ_026	JN092318
UN0027	499	BankIt1458394 SEQ_027	JN092319
UN0028	499	BankIt1458394 SEQ_028	JN092320
UN0034	498	BankIt1458394 SEQ_034	JN092326
UN0035	498	BankIt1458394 SEQ_035	JN092327

* Not available due to incomplete annotation.

† Serotype names replace the unique accession number following multiple confirmations and agreement between methods.

**Table 3:** Primers used to correlate genotype to serotype of *Salmonella enterica* by ISR

Primer name	Orientation	Primer sequence (5′–3′)	Reference	Amplicon size (bp)*
ISR-F1	Forward	GCCAATGGCACTGCCCGGTA	This study	14641
ISR-R1	Reverse	TACCGTGCGCTTTCGCCCAG		
ISRH-1	Forward	GATGCGTTGAGCTAACCGGTACTA	Morales *et al*. (2006)	Does not apply
ISRH-2	Reverse	ATTCTTCGACAGACACGGCATCAC		
ISRHfs	Forward	GTGGAGCGGTAGTTCAGTTGGTTA	Morales *et al*. (2006)	Does not apply
ISRHrs	Reverse	TAACCAACTGAACTACCGCTCCAC		

* Amplicon size in *S*. Typhimurium str. LT2 genome (GenBank AE006468).

For primer amplification, DNA was extracted from 1 mL of pelleted cells using the PureLink Genomic DNA Mini Kit (Invitrogen Life Technologies, Grand Island, NY). One microliter of DNA was added to 2× Gene Amp Fast PCR Master Mix (Applied Biosystems, Foster City, CA) and 200 nM forward (ISR-F1) and reverse (ISR-R1) primers in a final volume of 30 μL. The PCR was performed on a Veriti 96 well Fast Thermal cycler (Applied Biosystems) as follows: 95 °C for 10 s, 35 cycles at 94 °C, 40 s at 64 °C, and 10 s at 72 °C. After confirmation of the predicted amplicon of approximately 1400 bp by gel electrophoresis, PCR products were purified using the QIAquick PCR purification kit (Qiagen, Valencia, CA). DNA concentrations were measured (NanoDrop, Wilmington, DE) prior to submitting PCR products for Sanger sequencing (Retrogen Inc., San Diego, CA) on an Applied Biosystems, Incorporated (ABI) Prism® 3730 DNA Analyzer using primers ISRHfs and ISRHfr.

### Analysis and naming of ISR sequences

ISR sequences were aligned using SeqMan Pro of the Lasergene 8 software (DNASTAR, Madison, WI). Parameters were set to 100% minimum match percentage and a match size of at least 50 bp. Primers were designed to assure linkage to *dkgB*, which is required to assure that the correct region is under investigation. Serotype names were assigned to an ISR sequence when a 100% match was made to a reference sequence or when DNAhyb and KW serotyping agreed. Otherwise, ISR sequences are identified as ‘UN’ followed by four-digit numbers. The initial (5′ ATGTTTTGGCG 3′) and final (5′ CGGTGGAGCGG 3′) eleven nucleotides should be similar for ISRs, with the exception that the first nucleotide in the ISR sequence can sometimes be a cytosine rather than an adenine nucleotide.

### DNAhyb assay

The DNAhyb protocol was performed as directed (Check & Trace) on single *Salmonella* colonies grown for 24–48 h on BG agar at 37 °C. Large colonies are recommended to reach the recommended DNA concentration. Images of products were obtained on a single-channel ATR03 reader and processed by the *Salmonella* Check-Points software which indicates a serotype name, or alternatively, a genovar number. Images of spot patterns were discussed with the manufacturer in unusual situations, such as finding genetic variants of *Salmonella* Kentucky. In this case, three isolates were submitted to the source of the kit for independent verification that new variants were being identified and that all positive and negative controls worked.

## Results

### ISR and DNAhyb assign serotype to *S*. *enterica* similarly

Details from analysis of *S. enterica* by ISR and DNAhyb are shown in Table 1. Of the 139 submissions, 115 (82.7%) had substantial agreement between DNAhyb and ISR, as well as the reported KW serotype (Table 1a). Some genetic variation was noted in ISRs in this grouping, but serotype association was maintained in comparison with both DNAhyb and the KW scheme and thus these were counted as agreements. Further analysis of ISR variation showed that 15 named serotypes had at least three independent submissions. Of this group, 10 (66.7%) had uniform ISRs with no variation. Five (5) serotypes had multiple ISRs, namely *Salmonella* Infantis (two variants), *Salmonella* Typhimurium (two variants), *S.* Kentucky (two variants), *Salmonella* Newport (four variants), and *Salmonella* Montevideo (three variants). For the purpose of determining specificity and sensitivity in this study, results with disagreement about how much variation is accounted for by DNAhyb vs. ISR were counted as true negatives (TN), because the ISR method produced information somewhat different, but not necessarily in disagreement, to DNAhyb. In summary, 128 of the 139 isolates (92.1%) had agreement between DNAhyb and ISR (Table 1b + 1a). Detail about ISR variation within a single named serotype will be discussed in following text.

For 10 submissions, the forward/reverse (F/R) sequences did not match. Individually processing multiple colonies for nine of these submissions in this category recovered a second serotype. The frequency with which a second serotype accounted for disagreement between genomic methods and KW serotype suggests that minority subpopulations are common. In addition, techniques appear to vary in their ability to detect multiple serotypes. Seven other submissions (25011, 25048, 25040, 25041, 25035, 26026, and 100304.53) had KW serotypes with O-antigens that did not match what was received for analysis but there was no disagreement in F/R sequences. In these cases, misinterpretation of the second cell-surface molecule flagella could have contributed to misinterpretation of serotype. Alternatively, these submissions could have also been mixed when initially examined for KW serotype and undergone separation of serotypes prior to analysis during the current study. For submissions 101116-10 and 101116-12, which were classified as *Salmonella* Fresno and ISR UN0019, O-antigen D2 epitopes would be expected to cross-react with factor 9 antisera used to detect D1 epitopes (Curd *et al*., 1998).

Table 1c shows paired samples with disagreement between DNAhyb and ISR. Four of these submissions yielded mixed serotypes, namely 25043-1, 100723.01-1, 100723.02-1, and 100723-14.1. They are included in Table 1c to reflect the incidence with which disagreement was encountered. However, disagreements between DNAhyb and ISR and KW were resolved for these four isolates. The other seven submissions in Table 1c had a unique (UN) ISR sequence that was identified by DNAhyb as a genovar with an available reference sequence. However, slight differences in the ISR suggested genetic variants could be present as observed for the first group in Table 1a. These submissions were grouped in Table 1c because the serovar they might be associated with could not be further verified at this time. Acquisition of more isolates is needed to resolve the relationship between KW serotype, DNAhyb and ISR sequence in these cases.

### ISR appears more sensitive than DNAhyb for the detection of genetic variation within serotype

**Fig. 2:**
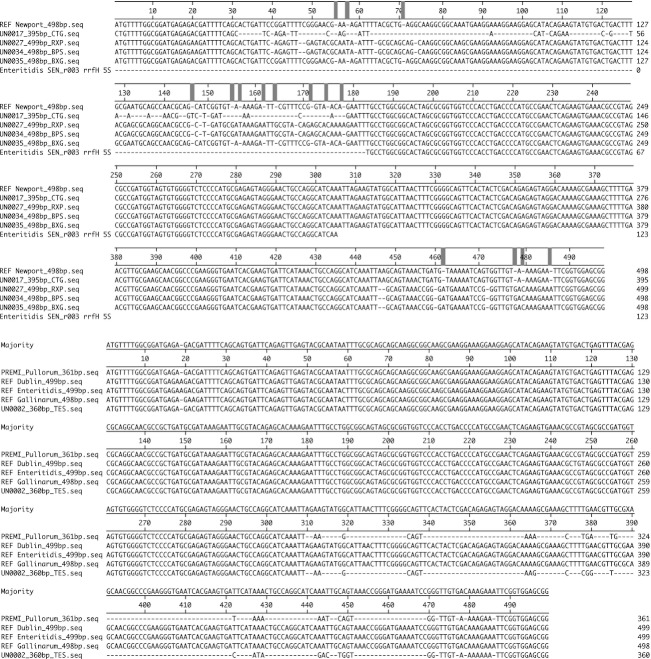
Alignment of ISR sequences to evaluate variation within serotype. (Top) Alignment of ISRs from *Salmonella enterica* serovar Newport. The shortest ISR shown is UN0017, which had a deletion occurring before the 5S ribosomal gene. The 5S ribosomal gene of *Salmonella* Enteritidis was included for reference purposes. (Bottom) Alignment of ISRs from the O-antigen group D serotypes of *S. enterica*. The submission with ISR UN0002 was *S*. Enteritidis by the KW scheme and Genovar 6660 by DNAhyb. ISR UN0008 was *Salmonella* Pullorum by the KW scheme and Genovar Gallinarum Pullorum 2978H by DNAhyb. Alignment of ISR sequences from Group D serotypes indicated that UN0002 is more like *S*. Pullorum. ISR UN0008 is more like *S*. Gallinarum or *S*. Enteritidis, except that it has a 146 bp insertion also found in *S*. Newport.

ISR appears to give a more sensitive assessment of serotype than DNAhyb. To explain further, some specific examples are cited. These are as follows:

1. ISR sometimes detected two types of *S*. Typhimurium, namely Typhimurium and Typhimurium 5-, whereas DNAhyb did not (Table 1, accession numbers 100304.57, 100304.74, 100304.32, 100304.62-2, 100616.9, 99163, 99164 and 99172). Thus, DNAhyb is not currently capable of detecting the 5- variant. ISR indicated that UN0014 was linked to the Typhimurium 5- KW serotype, but this correlation was not observed for all examples (see 100304.62-2). Thus, variation in expression of cell-surface epitopes may account for the 5- variant in addition to genetic variation as observed by ISR.
2. Only one publicly available reference sequence out of 27 disagreed with assignment of serotyping by the three methods. The ISRs for the reference sequences of *S*. Schwarzengrund NC_011094 and NZ_ABEJ00000000 were 267 bp and no SNP differences were present. The strains of *S*. Schwarzengrund analyzed here had ISRs of 257 bp regardless of method used to assign serotype. Further analysis is required to explain this discrepancy, which could be due to strain variation or some discrepancy with annotation in the reference strain.
3. Five submissions of *S*. Newport had different ISR sequences and four DNAhyb patterns despite having a single serotype assigned by the KW scheme. Alignment of the sequence of five ISRs from submissions that serotyped as *S.* Newport showed that ISR UN0017 had a deletion within the intergenic region between the end of the 23S and 5S rRNA genes (Fig. 2, Top).
4. O-antigen group D submissions had an even more complex ISR outcome than did *S*. Newport (Fig. 2, Bottom). Alignment showed that, in comparison with *S*. Enteritidis, ISR UN0002 had a somewhat similar deletion as that seen occurring within *Salmonella* Pullorum in the intergenic region between the 5S rRNA and tRNA *aspU*. In this same region, ISR UN0008 has an insertion that was 100% similar to a region from *S*. Newport found in a different ribosomal region. The insert accounted for the exceptional length of the ISR. Specifically, there was a 146 bp insert in ISR UN0008 that was the same as base pairs 4165975-4166120 in the genome of *S*. Newport strain SL254 (NC_011080). Other serotypes that had ISR variants within a serotype included *S*. Kentucky, *S*. Montevideo, and *S*. Infantis.

### ISR and DNAhyb are limited to assignment of serotype to *S. enterica*

The limit of detection of ISR was found by analysis of *S*. Enteritidis submissions 22079, 21027, and 21046, which were included to as control strains because they were previously characterized by whole-genome analysis (Guard *et al*., 2011). Strains 21027 and 21046 are clonally related and are within the same phage type lineage 13a/8, whereas 22079 is phage type 4. Despite belonging to the same phage type, strains 21027 and 21046 are phenotypically distinct and are known to have 16 genes with altered open reading frames as well as other SNPs. All three subpopulations of *S*. Enteritidis had the same KW serotype, DNAhyb genovar, and ISR. Thus, the two DNA methods were equivalent in regards to determining serotype only.

### Determination of sensitivity and specificity of ISR in comparison with DNAhyb

Table 1 includes the category of each ISR for the purposes of determining sensitivity and specificity in comparison with DNAhyb. True positives (TP) were defined as submissions assigned a serotype by ISR in complete agreement with DNAhyb, TN indicated ISR was different for the serotype assigned by DNAhyb due to the presence of genetic variation (including mixtures of serotypes), false positives (FP) were assigned a serotype by ISR but not by DNAhyb, and false negatives (FN) should have returned an ISR sequence but did not. As all submissions were *S. enterica* ssp. I and produced an ISR and a DNAhyb genovar, the FN value is 0. Calculating sensitivity from the values 124/124 + 0 (TP/TP + FN) suggests unity (similar performance) of the two methods. Calculating specificity from the values 8/7 + 8 = 0.53 (TN/FP + TN) suggests that DNAhyb is more specific, or in other words, it detects less genetic variation than does ISR. Removing submissions with mixtures did not change the finding that ISR appears to give more specific information than DNAhyb. Given that detection of new serotypes is a continuous process for *S. enterica* ssp. I, application of ISR has the potential to expand knowledge about diversity of serotypes. In these analyses, we used *S. enterica* serovar Enteritidis to provide a crucial control that shows ISR does not provide fine-scale differentiation achieved with whole-genome sequencing.

## Discussion

A limit of detection of ISR is that it targets a single region of the bacterial chromosome. Homologous recombination and other genomic events that mobilize DNA could generate a hybrid strain with potential to alter the correlation between an ISR region and the rest of the chromosome (Porwollik & McClelland, 2003). Methods that target multiple regions around the bacterial chromosome, such as DNAhyb and whole-genome sequencing, will thus still be required for critical stages of analysis. The primary use proposed for ISR is to facilitate routine and inexpensive serotyping of *S. enterica*. The method has been applied to processing DNA samples from South America in cooperation with the United States, and further development of software that incorporates a validated database will streamline analysis for users (Pulido-Landinez *et al*., 2012). SNP analysis by ISR complements methods such as DNAhyb that evaluate the whole genome, and each genome method can be used to check the quality of results from the other.

Disagreement between the KW scheme and genotyping by either DNA method could be attributed to at least four causes with a biological or molecular explanation.

1. Flagella H-antigen immunoreactivity may contribute disproportionately to interpretive differences between investigators;
2. A genetic variant may have a unique ISR or DNAhyb genovar that, in consensus with previous knowledge, is a genetic variant of an existing serotype;
3. Mixtures of serotypes could be present within cultures, which can be detected by some methods but not others.
4. The most troublesome group was new variants with undefined relationships to named serotypes. ISR UN0002 (ISR 360 bp) and UN0008 (ISR 530 bp) were associated with submissions serotyped by the KW scheme as *S*. Enteritidis and *S*. Pullorum, respectively, despite their unique ISR sequences. Classifying them by the KW scheme as *S*. Enteritidis or *S*. Pullorum could have unintended consequences, because the biological impact of these strains on susceptible hosts is not known. For example, *S*. Pullorum on-farm can initiate depopulation of chickens in order to protect poultry health (http://www.aphis.usda.gov/animal_health/animal_dis_spec/poultry/), whereas *S*. Enteritidis in people and foods can initiate control measures to protect human health (http://www.fda.gov/Food/FoodSafety/Product-SpecificInformation/EggSafety/EggSafetyActionPlan/ucm170746.htm). No information is available on the comparative virulence properties of UN0002 (ISR 360 bp) and UN0008 (530 bp) to *S.* Pullorum (ISR 361 bp) or *S*. Enteritidis (ISR 499 bp). Further research using biological assays is needed to characterize the virulence of strains identified by ISR as being potentially new strains of concern to either human or animal health.

Assay costs were from $10 to $12 per sample for ISR, $35 to $185 for KW serotyping and $50 for the method of DNAhyb used here. The point of comparison begins when a colony is identified on agar that is suspected of being *Salmonella*. The low cost and simplicity of conducting ISR make it a method that supports public health laboratories and food producers with in-house laboratories in their efforts to monitor *S. enterica*. Other efficiencies such as submission of DNA to centralized facilities and applying robotics for sample preparation may lower the cost of conducting ISR further. If a simple method for serotyping *S. enterica* is available, farm management and plant processors may test samples and monitor environments more frequently. The ability of ISR to detect a mixture of serotypes, its independence of cell-surface epitopes, cost, and simple software requirements are relative strengths. We suggest that it will a useful addition for assigning serotype at minimal cost rather than being another typing method with no clear advantage (Achtman, 1996; Achtman *et al*., 2012).

## References

[b1] Achtman M (1996). A surfeit of YATMs?. J Clin Microbiol.

[b2] Achtman M, Wain J, Weill FX (2012). Multilocus sequence typing as a replacement for serotyping in *Salmonella enterica*. PLoS Pathog.

[b3] Bopp C, Brenner FW, Wells J, Strokbine N, Murray P, Baron E, Pfaller M, Tenover F, Yolken R (1999). *Escherichia**Shigella*, and *Salmonella*. Manual of Clinical Microbiology.

[b4] Brenner FW, Villar RG, Angulo FJ, Tauxe R, Swaminathan B (2000). *Salmonella* nomenclature. J Clin Microbiol.

[b4000] Curd H, Liu D, Reeves PR (1998). Relationships among the O-antigen gene clusters of *Salmonella enterica* groups B, D1, D2, and D3. J Bacteriol.

[b5] Foodnet (2011). Vital signs: incidence and trends of infection with pathogens transmitted commonly through food–foodborne diseases active surveillance network, 10 U.S. sites, 1996–2010. MMWR Morb Mortal Wkly Rep.

[b6] Guard J, Morales CA, Fedorka-Cray P, Gast RK (2011). Single nucleotide polymorphisms that differentiate two subpopulations of *Salmonella* Enteritidis within phage type. BMC Res Notes.

[b7] Kauffman F, Edwards P (1952). A simplification of the Kauffmann-White schema. Am J Clin Path.

[b8] Madajczak G, Szych J (2010). Evaluation of PremiTest *Salmonella* kit for identification of not-typable by conventional methods *Salmonella*. Med Dosw Mikrobiol.

[b9] Malorny B, Guerra B, Zeltz P, Rissler K, Helmuth R (2003). Typing of *Salmonella* by DNA-microarrays. Berl Munch Tierarztl Wochenschr.

[b10] Morales CA, Gast R, Guard-Bouldin J (2006). Linkage of avian and reproductive tract tropism with sequence divergence adjacent to the 5S ribosomal subunit *rrfH* of *Salmonella enterica*. FEMS Microbiol Lett.

[b11] Popoff M, Le Minor L (2001). Antigenic Formulas of the Salmonella Serovars.

[b12] Porwollik S, McClelland M (2003). Lateral gene transfer in *Salmonella*. Microbes Infect.

[b13] Pulido-Landinez M, Laviniki V, Sanchez-Ingunza R, Guard J, Pinheiro do Nascimento V (2012). Use of FTA cards for the transport of DNA samples of *Salmonella* spp. from poultry products from southern Brazil. Acta Sci Vet.

[b14] Wattiau P, Van Hessche M, Schlicker C, Vander Veken H, Imberechts H (2008). Comparison of classical serotyping and PremiTest assay for routine identification of common *Salmonella enterica* serovars. J Clin Microbiol.

[b15] Wattiau P, Boland C, Bertrand S (2011). Methodologies for *Salmonella enterica* subsp. *enterica* subtyping: gold standards and alternatives. Appl Environ Microbiol.

